# A Multimodal Omics Framework to Empower Target Discovery for Cardiovascular Regeneration

**DOI:** 10.1007/s10557-023-07484-7

**Published:** 2023-07-08

**Authors:** Ziwen Li, Mairi Brittan, Nicholas L. Mills

**Affiliations:** BHF Centre for Cardiovascular Science, The Queen’s Medical Research Institute, University of Edinburgh, Edinburgh, UK

**Keywords:** Single-cell RNA sequencing, Myocardial infarction, Heart failure, Target discovery, Cardiovascular regeneration

## Abstract

Ischaemic heart disease is a global healthcare challenge with high morbidity and mortality. Early revascularisation in acute myocardial infarction has improved survival; however, limited regenerative capacity and microvascular dysfunction often lead to impaired function and the development of heart failure. New mechanistic insights are required to identify robust targets for the development of novel strategies to promote regeneration. Single-cell RNA sequencing (scRNA-seq) has enabled profiling and analysis of the transcriptomes of individual cells at high resolution. Applications of scRNA-seq have generated single-cell atlases for multiple species, revealed distinct cellular compositions for different regions of the heart, and defined multiple mechanisms involved in myocardial injury-induced regeneration. In this review, we summarise findings from studies of healthy and injured hearts in multiple species and spanning different developmental stages. Based on this transformative technology, we propose a multi-species, multi-omics, meta-analysis framework to drive the discovery of new targets to promote cardiovascular regeneration.

## Introduction

Heart failure due to ischemic heart disease is associated with a high morbidity and mortality despite treatment [1]. Early restoration of blood flow by percutaneous coronary intervention has improved survival in acute myocardial infarction. However, myocardial hypoperfusion or “no-flow” phenomenon [2] caused by microvascular dysfunction is common and can impact the effectiveness of revascularisation, limiting tissue regeneration and resulting in irreversible injury and heart failure [3, 4]. With the ageing of the population and increasing availability of early treatment for acute myocardial infarction, the prevalence of heart failure is projected to rise worldwide [5]. Numerous approaches, including pluripotent/stem cell transplantation, growth factor administration, and direct cell reprogramming, have been developed and tested in preclinical studies and clinical trials to promote tissue regeneration following ischemic injury, but the resulting benefits are transient and minimal [6]. New insights are required to broaden our understanding of the mechanisms underpinning endogenous cardiac regeneration and to develop more effective therapeutic strategies.

Human hearts, like most mammalian hearts, rapidly lose the ability to regenerate in the first week after birth [7–9]. This has presented a major challenge to regenerative medicine and has prompted researchers to either venture beyond mammals, studying species with superior regenerative capacity [10–12], or to turn back the clock, focusing on regenerative programs possessed by prenatal and early postnatal mammalian hearts. Furthermore, myocardial repair after infarction is a multi-stage process, involving inflammation, necrosis, angiogenesis, fibrosis, long-term remodelling, and maturation [13, 14], demanding a comprehensive regenerative strategy suitable for different stages of recovery.

Previous attempts to understand the mechanisms of myocardial repair and regeneration using traditional methods, such as quantitative PCR, bulk-RNA sequencing, flow-cytometry, and immunofluorescent staining, have generated invaluable insights into the transcriptional and translational change in major cell populations as a whole, but our understanding of heterogeneity and plasticity of individual cells and cell subpopulations has remained limited. The breakthrough in whole transcriptome analysis in single cells since 2009 [15] has ushered in a new era of precision in our understanding of cell biology and disease [16]. Building on the unprecedented resolution offered by single-cell technologies, the rise of spatial transcriptomics has further empowered the interrogation of gene expression by individual cells through the provision of their “geographical coordinates” within the tissue of interest [17]. Recent applications of these technologies have identified the dynamic transcriptomic signatures of major cell populations and novel subpopulations involved in cardiovascular development and in adult heart homeostasis and the pathogenesis of ischaemic heart disease [18, 19]. This presents an exciting opportunity for researchers to thoroughly map the microenvironment composition and biochemical cues and discover novel therapeutic targets that may elicit regenerative responses in the diseased heart [20].

This review will discuss the latest findings using these new sequencing technologies and propose a multi-species, multi-omics, meta-analysis framework to drive the discovery of new targets to promote cardiovascular regeneration.

## Understanding Cardiovascular Regeneration from a Single-Cell Perspective

Single-cell RNA sequencing (scRNA-seq) has been widely adopted to detect and quantify RNA molecules in individual cells, surpassing the traditional bulk-RNA-seq technology where a large number of cells are pooled and heterogeneity assessment is lacking [21]. The way we investigate organisms, organs, and tissues is transformed. Researchers have started building large-scale multi-organ single-cell transcriptomic atlases for different species such as Tabula Sapiens [22] and Tabula Muris [23] at an unprecedented speed. Cross-organ studies assessing major cell types, such as endothelial cells [24], smooth muscle cells [25], and fibroblasts [26], have generated valuable insights into how cells adapt to functional requirements at various anatomical locations. Facing ongoing healthcare challenges like cardiovascular disease and cancer [27–29] and public health crisis like the COVID-19 pandemic [30, 31], scientists have been able to quickly pinpoint the impacted cell types and transcriptional perturbations using scRNA-seq. Its wide application has led to the discovery of new lineage trajectories, cell populations and states, cell-cell interaction patterns, disease-specific transcriptional signatures, and potential therapeutic targets in a series of state-of-the-art studies in the cardiovascular field.

**Fig. 1:**
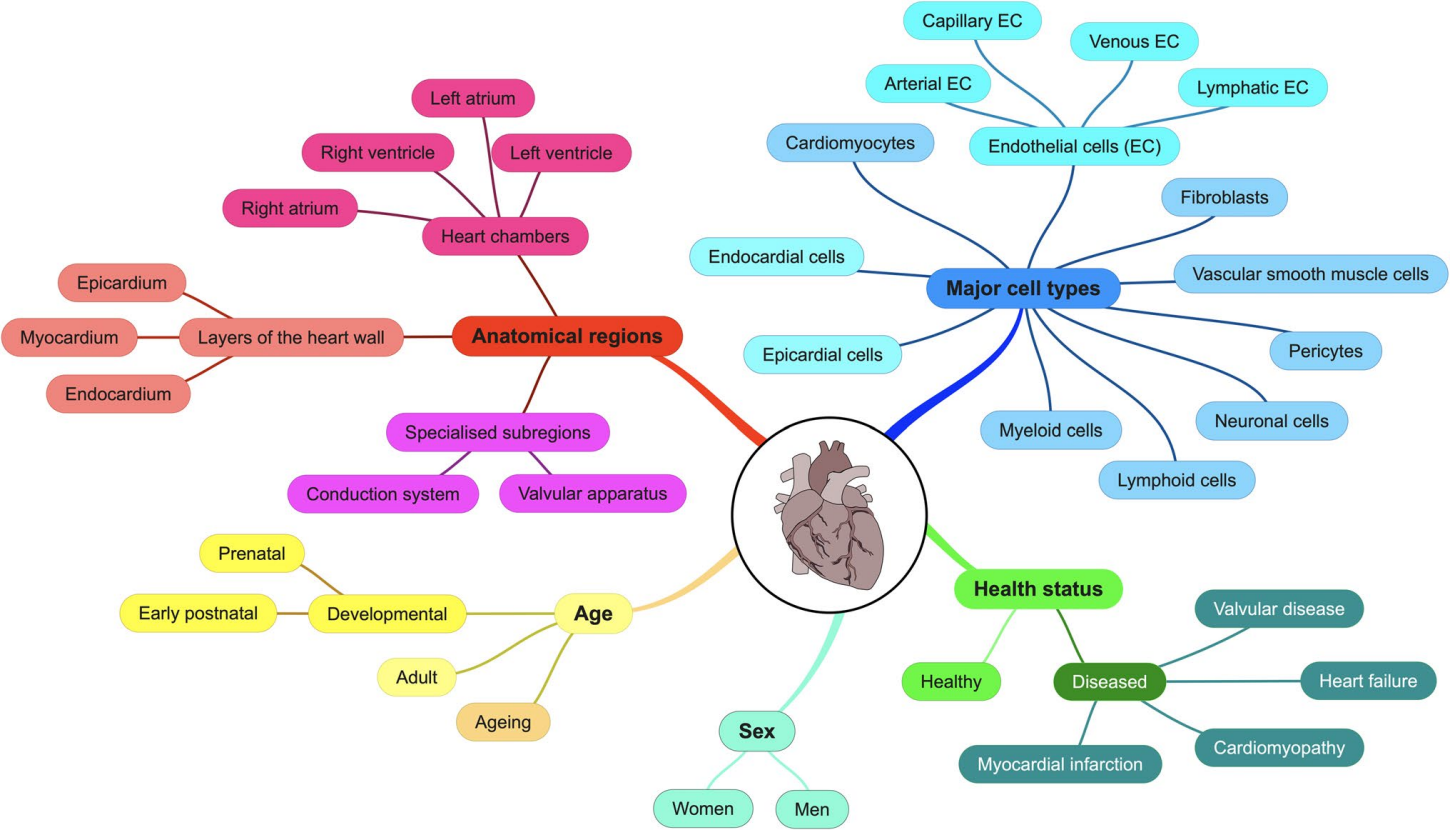
Map of factors to consider for single-cell studies in the cardiovascular field. The heart is a complex organ, and single-cell profiling studies focusing on specific questions often only provide limited information. This road map highlights some of the primary systems and factors that are now important to consider in a holistic manner to improve our understanding of heart function, repair, and regeneration. These systems and factors include different anatomical regions in the heart (the four chambers, the three layers of the heart wall, and specialised subregions like the conduction system and valvular apparatus), major cardiac cell types (endocardial cells, epicardial cells, cardiomyocytes, endothelial cells, fibroblasts, vascular smooth muscle cells, pericytes, neuronal cells, lymphoid cells, and myeloid cells), the health status of the heart (healthy or diseased such as myocardial infarction, cardiomyopathy, heart failure, valvular disease), sex (women and men), and age (prenatal, postnatal, adult, ageing)

The heart is a complex and dynamic organ. Profiling such an organ requires a careful review of the factors that shape the research questions, including (i) the anatomical region of interest, such as different heart chambers, layers of the heart wall, or specialised subregions like valvular apparatus; (ii) the major cell types to focus on, cardiomyocytes, endothelial cells, fibroblasts, etc.; (iii) the age of the heart, developmental, adult, or ageing; (iv) sex of the donor; and (v) status of the heart, whether it is healthy or diseased, such as myocardial infarction, cardiomyopathy, or heart failure. The studies included in this review have investigated some or a combination of these factors. We will review these studies and assimilate the findings in a holistic manner using the factors as a guide (Fig. 1).

### Surveying Cardiac Anatomy at Single-Cell Level

The first endeavour to build a comprehensive human heart atlas is marked by the study carried out by Litviňuková and colleagues, in which they identified eleven major cell types including atrial cardiomyocytes, ventricular cardiomyocytes, fibroblasts, endothelial cells, pericytes, smooth muscle cells, immune cells, adipocytes, mesothelial cells, and neuronal cells, from 45,870 cells, 78,023 CD45+ enrich cells, and 363,213 nuclei [32]. A clear atrial-ventricular difference was found in the cell composition: atrial tissues contained 30.1% cardiomyocytes, 24.3% fibroblasts, and 12.2% endothelial cells, while ventricular tissues had more cardiomyocytes (49.2%), less fibroblasts (15.5%), and less endothelial cells (7.8%) [32]. The proportions of ventricular cardiomyocytes and fibroblasts were negatively correlated [32]. Tucker et al. showed that cardiomyocytes were the most diverse cell type between atria and ventricles, and the left-right difference was less pronounced [33]. In contrast, the endothelial cells were more distinct by sides (left versus right), with 220 differentially expressed genes for the left versus right comparison and only 43 differentially expressed genes for atrium versus ventricle [33]. Like the four chambers, the four cardiac valves were also shown to have distinct cellular composition: migratory valvular interstitial cells (VICs), mast cells, myofibroblasts, and shear stress-reactive valvular endothelial cells (VECs) were found in the aortic valve; shear stress-reactive VECs and mast cells were in the pulmonary valve; nitric oxide (NO)-associated VICs, protective VECs, and migratory anti-inflammatory T cells were found in tricuspid valve; and mast cells and myofibroblasts were in mitral valve [34]. Other specialised subregions such as sinoatrial node (SAN) have also been studied at single-cell level: a study of 718 SAN cells from 21 adult mice has identified *Vsnl1* as a potential sinoatrial node marker and defined four distinct transcriptional clusters [35].

The chamber wall has three layers. The inner endocardium consists of endothelial cells and connective tissue, lining the four chambers; the middle myocardium mainly contains cardiomyocytes, responsible for heart contraction; the outer epicardium covers the myocardium and is largely mesothelium [36]. The endocardium and epicardium are quiescent in the adult heart but are actively involved in cardiogenesis [37, 38]. A study of embryonic day (E) 11.5 mouse hearts showed that the endocardial-specific deletion of *Hand2* disrupted multiple endocardial signalling pathways and marker gene expression and caused congenital defects, including tricuspid atresia, double inlet left ventricle, and interventricular septal defects, suggesting that endocardial *Hand2* is required for cardiac morphogenesis [39]. In a study of E12.5 and E16.5 mouse hearts*,* researchers found that a group of *Slit2*+ epicardium-derived cells (EPDCs) emerged after epithelial-to-mesenchymal transition (EMT), functioning as guidepost cells for migrating endothelial cells. An epicardium-specific deletion of myocardin-related transcription factors *Mrtf-a* and *Mrtf-b* disrupted the expression of guidance molecules and led to the ectopic accumulation of endothelial cells on the epicardial surface, indicating that paracrine signals from EPDCs are required for endothelial pathfinding and linking EMT to endothelial cell positioning [40].

### Resolving Cellular Heterogeneity in the Infarct Heart

Cardiomyocytes, capillaries, and extracellular matrix (ECM) are the main components of the myocardium enabling muscle contraction. Capillaries guard the supply of oxygen and nutrients, and fibroblasts produce ECM and maintain the structural properties. In acute myocardial infarction, fibroblasts are activated by the invading immune cells and expand to stabilise the infarct region, while endothelial cells proliferate to support new vessel growth in the border zone to restore the blood flow and limit the infarct size. Angiogenesis and fibrosis are key processes in healing after ischaemic injury, which has prompted researchers to profile endothelial cells and fibroblasts in the healthy and injured hearts. Despite being transcriptionally and functionally distinct, endothelial cells (ECs) and fibroblasts (FBs) show an interesting degree of commonality.

Firstly, both ECs and FBs have high heterogeneity, which changes with age. Litviňuková and colleagues identified ten EC populations in the adult human heart, including three capillary clusters (expressing *RGCC* and *CA4*, representing 57.4% of all ECs), one immune regulation–associated cluster (expressing *CX3CL1* and *IL6*), one arterial cluster (expressing *SEMA3G* and *EFNB2*), one venous cluster (expressing *NR2F2* and *ACKR1*), one atrial cluster (expressing *SMOC1* and *NPR3*, likely being endocardial cells), one lymphatic cluster (expressing *PROX1* and *PDPN*), and two clusters with fibroblast- and cardiomyocyte-like features respectively [32]. They also identified seven populations of FBs with an enriched expression of *DCN*, *GSN*, and *PDGFRA*, among which FB3 cluster expressed a high level of cytokine receptors, FB4 cluster expressed *POSTN* and *TNC* responsive to TGFβ signalling, FB5 cluster expressed genes related to ECM modulation, FB6 and FB7 showed endothelial- and cardiomyocyte-like features respectively [32]. By comparing the hearts from young adult (3 months) and aged mice (24 months), researchers discovered parallel alterations in age-affected circulating factors and endothelial expressed receptors and identified an apelin receptor–enriched endothelial subpopulation that was reduced with age [41]. Another study comparing the adult (3 months old) and ageing (18 months old) hearts further showed that ageing predominantly affected fibroblasts among other cell types, and aged fibroblasts had a higher transcriptional heterogeneity, with increased signatures in angiogenesis, inflammatory responses, and osteoblast differentiation, compared to the young fibroblasts [42]. To further assess whether the paracrine activity also altered with age in the fibroblasts, the study compared conditioned medium derived from aged and young mouse cardiac fibroblasts in vitro and found that the conditioned medium from the aged heart did not affect proliferation but impaired tube formation, enhanced pro-inflammatory activities, and reduced autophagy in endothelial cells [42].

Secondly, ECs and FBs both have a complex developmental origin, which has little impact on injury response. A study of the developing mouse and human hearts revealed that the transcriptional heterogeneity of coronary endothelial cells was initially influenced by lineage, originated from endocardium or sinus venous and then by anatomical location, until it declined in the homeostatic adult heart, and endothelial cells in the adult heart had similar responses to injuries regardless of their origins [43]. Similarly, most fibroblasts in the adult heart were derived from the epicardium, with two small fractions from endothelial cells and Pax3-expressing cells respectively, but the expansion of fibroblasts after injury was consistent regardless of their developmental lineage [44].

Thirdly, both ECs and FBs have small subpopulations in the steady state that can expand rapidly upon activation after ischaemic injury. A previous study of the healthy and 7-day post-myocardial infarction mouse heart demonstrated that clonal proliferation of resident endothelial cells sustained the endothelial structure after infarction and established the first endothelial atlas in the infarcted adult heart [45]. We defined ten endothelial clusters, including injury-specific clusters 6, 7, and 8 expressing high levels of *Plvap*. Immunofluorescence of Plvap/PLVAP in injured mouse and human heart tissues showed consistent upregulation at the protein level. The siRNA knockdown of *Plvap* significantly reduced endothelial proliferation in vitro, identifying Plvap as a potential angiogenic target [45]. Ruiz-Villalba and colleagues presented the first scRNA-seq study focusing on cardiac fibroblasts and showed that a subpopulation of the activated fibroblasts, expressing *Cthrc1*, expanded rapidly in response to myocardial infarction, from 2.3% at the steady state to 12% at 7-day post-injury and to 34% at 14 days, and these cells were exclusively located in the infarct and border zones, presenting a strong pro-fibrotic signature [46]. *Cthrc1* knockout mice showed a dramatically poorer survival rate after myocardial infarction (80% wildtype versus 30% knockout) due to ventricular rupture as a result of decreased collagen deposition [46]. The study also identified a population of fibroblasts with a similar transcriptomic profile in a swine myocardial infarction model and in the heart tissue from patients with myocardial infarction and dilated cardiomyopathy [46].

Finally, both ECs and FBs have subpopulations that can potentially take on a cardiomyocyte fate via transdifferentiation/reprogramming. It is worth noticing that in the human heart, both ECs and FBs have small subpopulations expressing cardiomyocyte markers [32]. We also identified a small disease-specific endothelial cluster that co-expressed cardiomyocyte marker *Tnnt2* in the injured mouse heart at 7-day post-myocardial infarction and confirmed its existence in the injured heart tissue using immunofluorescence [45], indicating a fate shift towards cardiomyocyte in response to injuries. Using an inducible fibroblast-specific *Tcf21-iCre*, Tani et al. overexpressed cardiac transcription factors Mef2c/Gata4/Tbx5/Hand2 in fibroblasts, which directly reprogrammed around 2% of cardiac fibroblasts into induced cardiomyocytes [47]. This was achieved by suppressing the expression of fibroblastic genes and converting pro-fibrotic cardiac fibroblasts to a quiescent anti-fibrotic state. In vivo reprogramming of cardiac fibroblasts using this technique has shown to improve cardiac function and reverse fibrosis in acute and chronic infarcted hearts [47].

Endothelial cells and fibroblasts play pivotal roles in angiogenesis and ECM modulation in the healthy and diseased hearts; however, the communication between the two has not been thoroughly investigated. Future studies on the in situ cross-talk between endothelial cells and fibroblasts will provide invaluable insights into their synergy in tissue repair and regeneration and inspire new therapeutic strategies that can simultaneously target both populations.

### Deciphering the Transcriptomic Complexity in the Failing Heart

The acute loss of myocardium and the resultant load increase following myocardial infarction triggers a cascade of biochemical signalling processes, which modulate post-injury repair and remodelling, involving dilation, hypertrophy, and scar formation [48]. Hypertrophy is an adaptive response to the increased load and progressive dilation during ventricular remodelling. In preclinical research, pressure overload hypertrophy is induced by transaortic banding (TAC) in mice. Ren et al. profiled the major cell types during the progression of pressure-induced hypertrophy at 0-, 2-, 5-, 8-, and 11-week post-TAC and described a stagewise change of cellular cross-talk [49]. They discovered that macrophage activation and subtype switching occurred at the middle stage (2–5 weeks), and fibroblasts and endothelial subtypes were correlated with disease progression especially at the late stage (5–11 weeks) [49]. Wang et al. studied 21,422 cells from the normal, failed, and partially recovered (left ventricular assist device treatment) adult human hearts and found that the contractility and metabolism of cardiomyocytes were prominently correlated with the changes in heart function [50]. They discovered that non-myocyte cells actively regulated myocyte behaviours and identified a subpopulation of *ACKR1+* endothelial cells that could preserve cardiac function after injury [50].

Heart failure is a complex clinical syndrome, encompassing a diverse group of clinical features converging on impaired muscle function. Recent studies have mapped the transcriptional and cellular diversity of human hearts with different types of cardiomyopathy, including hypertrophic cardiomyopathy (HCM) [51], dilated cardiomyopathy (DCM) [51–54], ischaemic cardiomyopathy (ICM) [52], and arrhythmogenic cardiomyopathy (ACM) [54]. Rao et al. profiled non-cardiomyocyte cells from late-stage DCM and ICM hearts and identified AEBP1 as a crucial regulator of cardiac fibrosis in ACTA2+ myofibroblasts [52]. They also discovered a CXCL8^hi^CCR2+HLA-Dr^hi^ macrophage subpopulation enriched in a severely fibrotic area, which interacted with endothelial cells and potentially facilitated leukocyte recruitment [52]. Koenig et al. defined the transcriptional landscape of 25 non-diseased and 13 non-ischaemic DCM left ventricles and investigated the impact of age, sex, and disease state on the transcriptomes [53]. They found that cell composition did change in DCM individuals but was not associated with the severity of heart failure, and no clear differences by sex were detected in the non-diseased or DCM group. They observed a converging trend in cardiomyocytes toward common disease-associated states, while fibroblasts and myeloid cells were increasingly diversified. Endothelial cells and pericytes showed global transcriptional shifts but little to no change in cell composition [53]. Chaffin and colleagues profiled the left ventricles from 11 DCM, 15 HCM, and 16 non-failing hearts and identified a large transcriptional change in DCM or HCM left ventricles compared to the non-failing ones, though few differences were found between DCM and HCM despite their distinct genetic bases [51]. They compared HCM samples with preserved left ventricular ejection fraction (LVEF) to HCM samples with reduced LVEF and found no convincing transcriptional differences in any cell types, indicating a convergence to a common transcriptional profile in advanced cardiomyopathy [51]. Reichart and colleagues sequenced the samples from the left ventricle free wall, apex, septum, and right ventricle free wall from non-ischaemic failing hearts with pathogenic variants in DCM and ACM and performed a genotype-stratified analysis of cell lineages and transcriptional states [54]. The cell atlas from DCM and ACM ventricles showed different left and right ventricular responses and demonstrated genotype-specific signalling pathways, cell-cell communications, and differentially expressed genes at single-cell levels. They further applied a machine learning approach and successfully developed a graph attention network classifier that was trained on the snRNA-seq data from four major left ventricular cell types (cardiomyocytes, fibroblasts, endothelial cells, and myeloid cells) and was able to predict the genotypes with high accuracy [54]. Simonson et al. performed snRNA-seq on non-infarcted samples from the left ventricles of patients with long-term ICM and observed a significant decrease in cardiomyocytes and an endothelial subpopulation expressing high *ARHGAP28* and *FBN1* [55]. Genes associated with proliferation and angiogenesis were enriched in endothelial cells, and lymphatic endothelial cells increased in all ICM samples [55]. By combining the HCM and DCM data from Chaffin et al. [51], Simonson and colleagues also identified the genes that were up- and down-regulated in ICM but not in HCM or DCM [55]. Furthermore, they compared the genes with consistent change across all three cardiomyopathies to the druggable genome [56], resulting in 39 druggable genes (10 up-regulated and 29 down-regulated) including *CYD2J2*, *MYH6*, *COL14A1*, and *SPARCL1* [55].

While these individual studies only investigated one or two types of cardiomyopathy, they have collectively encompassed the full spectrum of cardiomyopathy (Table 1). Four [51–54] out of the five studies have primarily analysed a total of 81 DCM samples, meriting further investigation into the shared characteristics. All four studies have profiled major cardiac cell types including cardiomyocytes (though not in Rao et al.), fibroblasts, endothelial cells, and macrophages. A decrease in cardiomyocytes was observed across all studies and in all genotypes except in the *LMNA* pathogenic variant (PV) carriers. An increase in fibroblasts was shown in all studies except Reichart et al. despite clear histopathological findings of fibrosis. Angiogenic signatures were up-regulated in endothelial cells across all studies, and a reduction in proliferating macrophages was observed in Koenig et al. and Chaffin et al. Both Chaffin et al. and Reichart et al. investigated the impact of *TTN* mutations. The former detected minimal transcriptional differences between the *TTN* loss-of-function mutation carriers and other DCM patients, while the latter observed both cell compositional and transcriptional changes that were specific to the *TTN* PV carriers. A high degree of agreement in the findings is readily appreciated in these studies; however, discrepancies and unanswered questions warrant follow-up investigation. Furthermore, subgroup analysis, such as a sex-specific profiling for the *TTN* PV carriers in Reichart et al., was not feasible due to limited sample size and would certainly benefit from incorporating external datasets, like the data from the *TTN* mutation carriers in Chaffin et al. A meta-analysis approach to integrate datasets and assimilate findings from individual studies would help ensure validity and stimulate new analyses as shown briefly above. In the process of curating the studies, knowledge/data gap could also be identified—more data from HCM, ICM, and ACM are clearly required here to obtain a complete map of the disease-specific transcriptome and to enable subgroup analyses stratified by age, sex, and genotype (Table 1).

**Table 1:** Overview of sample numbers and cell types in the cardiomyopathy studies [51–55]

Studies	HCM	DCM	ICM	ACM	Non-diseased heart	Cell types profiled
Rao et al. [52]	–	*N* = 5	*N* = 4	–	*N* = 2	Non-cardiomyocyte cells
Koenig et al. [53]	–	*N* = 13*	–	–	*N* = 25*	All cell types
Chaffin et al. [51]	*N* = 15	*N* = 11	–	–	*N* = 16	All cell types
Reichart et al. [54]	–	*N* = 52	–	*N* = 8	*N* = 18	All cell types
Simonson et al. [55]			*N* = 7		*N* = 8	All cell types
Total	*N* = 15	*N* = 81	*N* = 11	*N* = 8	*N* = 69	–

*HCM* hypertrophic cardiomyopathy, *DCM* dilated cardiomyopathy, *ICM* ischaemic cardiomyopathy, *ACM* arrhythmogenic cardiomyopathy, *N* number

*The referenced study reported that 27 healthy donors and 18 individuals with dilated cardiomyopathy were included. However, entries of only 38 patients were found in the supplementary patient metadata. The discrepancies are likely due to double counts of patients whose samples were processed using both snRNA-seq and scRNA-seq

### Harnessing Endogenous Regenerative Capacity in Non-mammalian Species

Unlike humans, salamanders and zebrafish [57] can regenerate their hearts throughout life, making excellent models for cardiovascular regeneration. After injuries, axolotl hearts can undergo macrophage-modulated cardiomyocyte proliferation to regenerate, and macrophage depletion leads to progressive scarring and impaired ventricular function [58]. An axolotl single-cell study revealed that axolotl hearts contained *Gata4*+ endothelial cells, supporting both angiogenesis and cardiomyocyte regeneration [59]. Using scRNA-seq, three epicardial subpopulations and seven cell clusters have been identified in the developing [60] and adult zebrafish hearts [61] respectively. Transcription factor prrx1b, expressed in the epicardial- and epicardium-derived cells, was shown required for scar-free zebrafish heart regeneration. Loss of prrx1b caused excessive fibrosis and impaired cardiomyocyte proliferation by tuning Nrg1 expression [62]. The transient activated epicardial progenitor cells (aEPCs), expressing ptx3a and col12a1b, underwent epithelial-to-mesenchymal transition (EMT) to differentiate into mural cells and mesenchymal cells. A conditional ablation of these aEPCs was shown to block heart regeneration through reduced Nrg1 expression and decreased mesenchymal population [63]. Heterogeneous pdgfrb+ epicardium-derived cells were also found essential for heart regeneration [64]. In the endocardium, absence of Runx1-positive cells promoted myocardial proliferation and protection [65]. A transient subpopulation of collagen-12-expressing fibroblasts, which originated from both the epicardium and endocardium, has proved proregenerative [65]. A cardiomyocyte-focused study in zebrafish has revealed that border zone cardiomyocytes underwent metabolic reprogramming induced by Nrg1/ErbB2 signalling after injuries, making them resemble embryonic cardiomyocytes [66]. Taken together, restoration of zebrafish and salamander hearts is accomplished through the efforts of multiple cell types and subpopulations via specific signalling pathways such as Nrg1. Further studies of the epicardium are required to gain a comprehensive understanding of its role in adult heart regeneration.

### Studying the Developmental Heart to Inform Adult Cardiovascular Regeneration Pathways

Adult mammalian hearts regenerate poorly, but their prenatal and early postnatal counterparts restore fully with minimal fibrosis and function normally [7–9]. These observations have led to the development of two reprogramming strategies: to reactivate the foetal regenerative programs and to deactivate the senescence programs in the adult heart. An analysis of the E9.5, E12.5, and postnatal (P)1 mouse cardiomyocytes has revealed a “progenitor-like” subpopulation that was abundant in the early embryonic mouse hearts but decreased to minimal postnatally [67]. The same study also found that epicardial progenitors were unlikely to be the source of cardiomyocytes using an epicardial lineage tracing model, as the labelled clones were primarily fibroblasts and smooth muscle cells [67]. The combined application of the FUCCI system and scRNA-seq has enabled direct comparisons between cycling and non-cycling cardiomyocytes at P0 (regenerative) and P7 (non-regenerative) and demonstrated that cycling cardiomyocytes had the highest mitotic rate at birth, which gradually declined during neonatal period [68]. The differentially up-regulated genes in cycling P0 cardiomyocytes included members of the Hippo/YAP and Nrg1/ErbB2 signalling pathways and genes required for cardiomyocyte cytokinesis like *Ect2* and *RhoA* [68]. Another study comparing the regenerative (P1) and non-regenerative (P8) neonatal hearts showed that many epicardial-enriched and injury-induced genes were specific to the regenerative hearts, among which RSPO1 was a key ligand mediating crosstalk between epicardial and endothelial cells and enhancing the angiogenic ability of endothelial cells [69]. In contrast to the regenerative hearts, epicardial cells from the non-regenerative hearts secreted pro-fibrotic factors post-injuries [69]. Epicardial-derived cells (EPDCs) are essential for cardiac regeneration during mammalian development [67, 69] and in lower vertebrates with superior regenerative capacity [60–64]. However, it is unclear how many of these cells exist and to what extent they can differentiate into other cardiac cell types to support regeneration in the adult human heart. Knight-Schrijver and colleagues have recently explored this uncharted space in a scRNA-seq study comparing the cellular composition and transcriptional states of foetal and adult human epicardium [70]. They found that the adult epicardium had a minimal population of mesenchymal EPDCs and reduced paracrine communication with other cardiac cell types and was lacking angiogenic epicardial programs including WNT signalling compared to their foetal counterpart [70]. They also revealed an age-associated transition of human epicardium from an angiogenic-responsive state toward an immune-responsive state, likely a previously overlooked element in understanding the role of immune response in cardiac regeneration. A recent study on aortic endothelial cells in 4-, 26-, and 86-week-old mice has shed light on senescence heterogeneity among different endothelial subpopulations, showing upregulation of senescence-associated genes *P53* and *P21* and downregulation of proliferation-associated genes *Ki67* in the Gpihbp1+ EC1 subpopulation, compared to the Plvap+ EC2 [71]. Taking a more direct approach, Chen et al. overexpressed the Yamanaka factors [72] Oct4, Sox2, c-Myc, and Klf4 in cardiomyocytes for 6 days before or during injuries and achieved the repair of amputated apex with minimal scarring at P7, a time point when cardiomyocytes normally stopped proliferating and the regenerative capacity of the heart is completely lost [73]. They successfully demonstrated that the transient dedifferentiation and reprogramming of adult cardiomyocytes could improve left ventricular function, provided it was initiated as early as possible post-myocardial infarction, and they also cautioned that the process carried risks of cellular transformation and neoplasm formation if not tightly controlled [73]. In summary, steady progresses have been made towards rejuvenating adult heart tissue to promote injury-induced repair, and more in-depth studies are required to develop and validate these approaches and explore their safety and efficacy.

### Multimodal Profiling of the Single-Cell Multiome

Turning back ageing clocks in the heart requires a deep understanding of epigenetic control of cardiovascular development, homeostasis, and diseases. Epigenetic changes accumulate during ageing [74], leading to disrupted gene expression, reduced tissue function, and diminished regenerative capacity. With technologies like scATAC-seq (assay for transposase-accessible chromatin using sequencing) and scMethyl-seq (DNA methylation sequencing), we can map the epigenetic landscape at single-cell levels and gain further insights into the transcriptional regulatory networks. Wang et al. compared the post-MI P1 and P8 mouse hearts using scATAC-seq and found that fibroblasts had the greatest number of both myocardial infarction-induced and infarction-repressed regions among the major cell types, and more myocardial infarction-responsive regions were found in the P8 hearts than in the P1 hearts, indicating a prominent open chromatin dynamics in fibroblasts concurrent with cardiac remodelling [69]. To assess the chromatin level change in the cardiomyocyte-like cells (iCMs) induced by reprogramming factors Mef2c/Gata4/Tbx5 (MGT), Wang and colleagues performed scATAC-seq on cardiac fibroblasts at 3 days post-MGT treatment and revealed several transcription factor regulatory networks associated with the conversion, including FOS-AP1, SMAD, and TEAD [75]. They showed that accessible regions with the motifs of FOS-AP1 were rapidly closed upon MGT induction, and SMAD family members exhibited diverse modulation roles on iCM reprogramming at different stages post-induction [75]. Single-cell profiling at the protein level has recently come into play. Schoof and colleagues presented the first large-scale single-cell mass spectrometry–based proteomics with comprehensive experiment and computational analysis workflow [76]. The novel approach allowed consistent quantification of 1000 proteins per cell across thousands of cells, and the companion software Sceptre could seamlessly normalise the data, integrate FACS data, and assist downstream analysis [76]. With the development and maturation of these new technologies, future studies can routinely perform simultaneous profiling of genome, epigenome, transcriptome, and proteome, covering the full genetic information flow from DNA to RNA to protein, providing comprehensive multi-omics map for healthy and diseased hearts at the single-cell level.

## Triangulating Injury Responses Using Spatial Coordinates

Single-cell sequencing technology relies on the successful isolation of individual cells [77], during which the spatial information of each cell is irreversibly lost. By combining single-cell and spatial transcriptomics, researchers have gained insights into the spatial distribution of different cell types and subtypes and how individual cells with specific transcriptional profiles function collectively with the surrounding cells in the microenvironment. Asp et al. constructed the first spatial transcriptomics map for the embryonic human hearts spanning three developmental stages, enabling studies of cardiac morphogenesis [78]. Misra and colleagues analysed P2 and P10 heart tissues post-apical resection with spatial transcriptomics, which defined the scar tissue and its transitioning from a proliferative to secretory phenotype as the heart lost regenerative capacity. They also found a regenerative border zone defined by immature cardiomyocyte markers such as *Ankrd1* and strong expression of cardioprotection factor *Sprr1a*. Kuppe et al. constructed the first spatial multi-omics map of human myocardial infarction and characterised tissues from the ischaemic zone, boarder zone, remote zone, and fibrotic zone using cell-type deconvolution [20]. Boileau et al. developed Single-cell Nanopore Spatial Transcriptomics (SCNAST) software, based on long-read sequencing data barcode assignment, to generate a full-length near-single-cell transcriptional landscape of the tissue microenvironment [79]. Using this method, they profiled four mouse heart sections at 3 days post-myocardial infarction and discovered previously uncharacterised modes of transcription, such as intron retention, which was associated with the infarct area. They also identified 109 significant regional isoform switching genes across all comparisons between the remote, border, and infarct areas [79].

## Difficulties in Connecting a Million Dots

Researchers have gained valuable insights into the many faceted nature of cardiac regeneration through numerous studies in a range of organism models over the years. However, this has not resolved into a clear picture of the mechanistic network governing regeneration in the adult human heart. It is largely due to failure in using small animal models to faithfully reproduce human disease and recapitulate human responses to interventions. After all, cardiac cellular composition varies among different species [32, 80, 81], and each species has unique regulatory programs in heart homeostasis and disease [82, 83]. The difference could sometimes preclude further development of drugs and therapies for reasons like the lack of human orthologous of a promising target gene discovered in mice [84]. Furthermore, the central dogma, from DNA to RNA and from RNA to protein, is at the centre of all biological processes, and each step also has many pre- and post-regulatory mechanisms. While DNA and RNA can cause structural and functional changes in protein, leading to phenotypic perturbations, disease-associated phenotypes are more often caused by altered regulatory mechanisms. Subsequently, inconsistency is observed between different layers of omics. Pei and colleagues profiled 13 HCM (with truncating *MYBPC3* mutations) and 10 control hearts using chromatin immunoprecipitation sequencing (ChIP-seq), RNA-seq, and proteomics [85]. They identified HCM-related changes in histone acetylation (4226 regions with higher acetylation and 5084 with lower acetylation), transcription (936 up-regulated genes and 1097 down-regulated genes), and translation (216 up-regulated proteins and 225 down-regulated proteins) [85]. Despite the rich information from each omics layer, only 5 genes including *ADPN*, *FMOD*, *MCAM*, *NPPA*, and *AASS* showed a consistent change in the same direction in the HCM versus control hearts at DNA, RNA, and protein levels [85], reflecting the complex relationship between different omics layers and cautioning us against drawing conclusions based on a single layer.

To better understand the mechanism and pinpoint the omics layer(s) to target for intervention at the single-cell level, researchers have started combining multiple single-cell omics technologies. Alexanian and colleagues performed scRNA-seq and scATAC-seq on the healthy and pressure overload mouse hearts and found that the fibroblasts had increased chromatin accessibility post-TAC [86]. They identified transcription factor (TF) MEOX1 as a central regulator of fibroblast activation and showed that *Meox1* knockdown reduced the formation of stress fibres and expression of *Acta2* in TGFβ-treated fibroblasts [86]. Ameen and colleagues also integrated scATAC-seq and scRNA-seq data from human foetal heart samples and mapped the landscapes of cis-regulatory elements (CREs) and genes that define major cell types during cardiogenesis [87]. They successfully trained deep learning models to interpret the cell-type-specific sequence of active TF binding sites, predict the impact of de novo non-coding mutations on chromatin accessibility, and infer the active TF binding sites disrupted by high-impact mutations [87]. They validated an endothelial-specific enhancer that harboured a predicted high-impact mutation related to *JARID2* using CRISPR/Cas9 deletion and showed reduced *JARID2* expression and depletion of tubes using a tube formation assay in iPSC-derived endothelial cells [87]. This analysis framework created a new way to study putative causal, de novo non-coding mutations associated with congenital heart diseases. Hocker and colleagues profiled ~80,000 cardiac cells using snATAC-seq on adult human heart tissues and created a comprehensive atlas of cardiac *cis*-regulatory elements (cCREs) [88]. By integrating snRNA-seq data from the matched samples, they identified differentially accessible cCREs underlying chamber- and cell-type-specific gene expression patterns and potential causal candidate cCREs involved in heart failure and atrial fibrillation (AF). They validated the role of one cCRE that contained AF risk variants in regulating the expression of potassium channel gene *KCNH2* and action potential repolarisation in hPSC cardiomyocytes, using CRISPR/Cas9 genome deletion [88].

On the one hand, it is increasingly difficult to effectively synthesise the knowledge from previous studies in the field; on the other hand, new technologies are generating more data and creating new avenues at unprecedented paces. Both require the creation of a framework to systematically and continuously assimilate findings from research studies to improve our understanding of the complex mechanism in cardiovascular diseases.

## A Multi-species Multi-omics Meta-analysis Framework

**Fig. 2:**
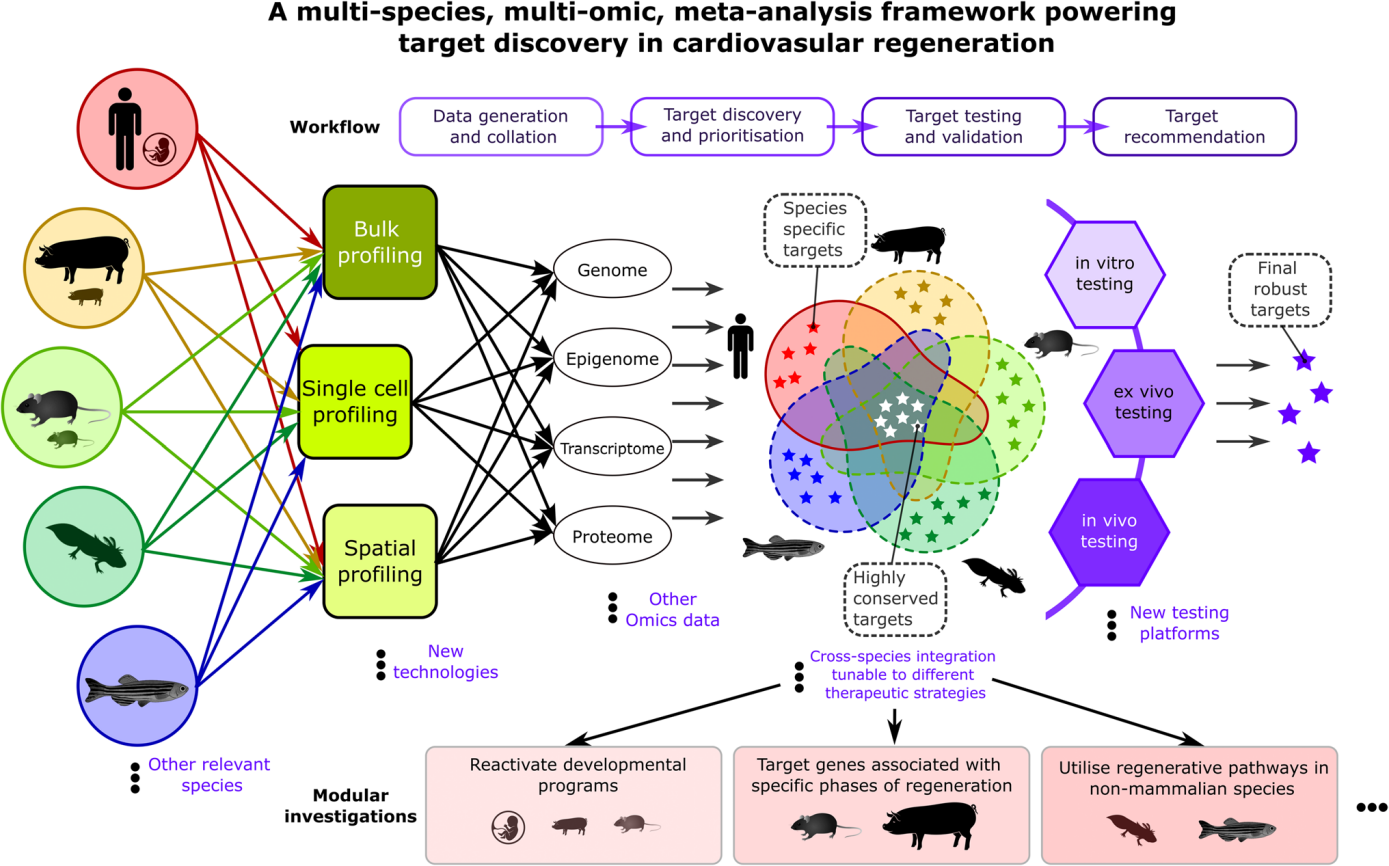
Schematic of a multi-species, multi-omics, meta-analysis framework for target discovery in cardiovascular regeneration. There are four main stages in the framework: stage 1, bulk/single-cell/spatial profiling of genome, epigenome, transcriptome, and proteome data from human, large animal models like porcine, small animal models like mice and rats, highly regenerative non-mammal models like axolotl and zebrafish, will be collated and inventorised based on species and three main technologies, including bulk, single-cell, and spatial profiling; stage 2, multi-omics data (including genome, epigenome, transcriptome, and proteome profiling) are integrated vertically within each species and technology, potential targets and their regulatory networks are identified, and targets from individual integrations are pulled out and prioritised based on the factors like expression level, relevance to human pathophysiology, and novelty; stage 3, top ranked targets are tested in in vitro, ex vivo, and finally in vivo experiments; stage 4, targets with the greatest safety and efficacy recommended for further preclinical or clinical investigations. The framework will embrace new technologies/omics data and knowledge from other species; allow modular investigation and selective integration tunable to specific therapeutic strategies, such as reactivating the developmental programs, targeting specific phases of the regeneration process, and utilising conserved regenerative pathways uniquely activated in non-mammalian species; and explore new testing platforms

Breaking through the barrier to heart regeneration in humans, we need to use a comprehensive approach and test all possibilities. We have previously carried out a meta-analysis of integrated scRNA-seq data from coronary endothelial cells extracted from injured and healthy developing adult mouse and human heart, which enabled us to gain new insights into the conserved pathways between the injured human and mouse hearts, unique regenerative programs used by the developing hearts, and transcriptional shifts in heart failure [89]. We identified VEGFC, KLF4, EGR1, and ZFP36 as potential new targets for vascular regeneration and generated a coronary endothelial cell meta-atlas CrescENDO Shiny application to empower future research [89]. By constantly integrating data from species with higher regenerative capacity like axolotls and zebrafish from other omics technology such as scATAC-seq and spatial transcriptomics, and disease data spanning various timepoints after myocardial infarction and different stages of heart failure, we will be able to construct an exhaustive database that allows fast knowledge base building and rapid target discovery, prioritisation, and validation. Therefore, we propose a multi-species, multi-omics, meta-analysis framework (Fig. 2), to assimilate the latest scientific discoveries, inform new mechanisms, and generate novel targets for successful cardiovascular regeneration. There are four main stages in this framework: data generation and collation, target discovery and prioritisation, target testing and validation, and target recommendation. In stage 1, previously published and newly generated bulk, single-cell, and spatial datasets will be collated for each species; in stage 2, targets will be pulled out from individual studies with their regulatory network mapped out with the multi-omics data, and these targets will subsequently be prioritised based on the expression level, relevance to patients, and novelty; in stage 3, top ranked targets are tested in vitro (cultured primary cells and organoids), ex vivo (cultured tissue slices and biopsies), and in vivo (small and large animal models); in stage 4, the performance of the targets are assessed, and the ones with the greatest safety and efficacy will be recommended for further preclinical or even clinical investigation. As the framework grows, it will embrace new technologies/omics data and knowledge from other species that are clinically relevant; allow modular investigation and selective integration tunable to different therapeutic strategies, such as reactivating the developmental programs, targeting specific phases of the regeneration process, and utilising conserved regenerative pathways uniquely activated in non-mammalian species; and explore new testing platforms as they become available.

Establishing and maintaining such a framework require the wider research community to work together and act collectively, coordinating studies, providing datasets, creating new pipelines for multimodal data, automating target prioritisation, optimising downstream testing pathways, fine-tuning recommendation algorithms, and building and updating knowledge base. This in turn will empower broader and more impactful international research, encouraging scientists from different disciplines to join forces to tackle the challenges in cardiovascular regeneration and accelerate therapeutic innovation. The Human Cell Atlas Consortium [90] and CZ CELLxGENE Discover [91] have demonstrated the feasibility of large-scale single-cell data curation, integration, annotation, and sharing. Learning from their success will be instrumental in the early stage of building this framework. Multimodal approach has been adopted by artificial intelligence (AI)-augmented drug discovery companies such as BenevolentAI to help understand disease biology, build data foundation, and inform new targets [92]. Collaborating with leading AI drug discovery companies will significantly accelerate the establishment and refinement of the framework.

## Understanding the Challenge Ahead

A fully functioning and well-maintained framework would serve as a powerful resource for the research community, but setting it up is not easy. A major challenge is multi-omics data integration, for unmatched (of the same modality but from different cells) and matched (from the same cells but of different modalities) datasets [93]. The former, also called horizontal integration, uses genes as the anchor for integration, while the latter, vertical integration, uses cells. Horizontal integration has been used for integrating large-scale scRNA-seq datasets generated across multiple batches and technologies. The tools for horizontal integration, such as Seurat v3 [94], Harmony [95], and scVI [96], aim to remove batch and other technical variances while preserving the biological differences. Vertical integration can be divided further into local integration, focusing on defining putative interaction across omics layers, and global integration, with an aim to understand broader cellular states like the cell cycle. Tools like MOFA [97] and Seurat v4 [98] are used for global integration and LMM [99] for local integration. To integrate datasets of different modalities and from different sets of cells, we need to perform diagonal integration, which is much more challenging because of no anchors, and the findings are often difficult to interpret. Diagonal integration is based on a critical assumption that similarities are preserved between different omics layers. One of the strategies for such integrations is to group cells into different cell types within each layer first, but this has its own assumption that the cell-type signatures are the same across omics layers, which is not always true. A recent integration tool GLUE (graph-linked unified embedding) explicitly modelled the regulatory interactions between omics layers and has shown to be more accurate and robust in challenging tasks like triple-omics integration [100].

The widely used specialised packages such as Seurat can handle certain integration tasks, but more machine/deep learning–based tools are required to resolve the interaction across omics layers to build a gene regulatory network. Meanwhile, we need to create gold-standard multi-omics datasets specifically for the heart and cardiovascular diseases, which will provide the ground truth for building and evaluating new multi-omics methods. Generating such datasets requires robust study design, careful assessment of statistical power and effect size, identification of confounders, biases, and sources of variation, and quality control. A good example of such datasets is The Cancer Genome Atlas Program (TCGA) dataset [101], which has curated 2.5 petabytes of genomic, epigenomic, transcriptomic, and proteomic data from over 20,000 primary cancer and matched normal samples spanning 33 cancer types since 2006. Many multi-omics integration tools are developed using the TCGA dataset, and further investigation is required to test whether these tools can be generalised beyond the cancer research setting and readily adopted in the cardiovascular field. CRAN also recommended a series of tools for multi-omics data integration and analysis in “CRAN Task View: Genomics, Proteomics, Metabolomics, Transcriptomics, and other Omics (Version 2023-04-03)” [102]. Time-series and spatial mapping studies add further complexity to the already challenging integration task. Luckily, solutions to predict how DNA, RNA, and protein co-vary in single cells using multi-omics time-series data have been demonstrated in the prediction competition “Open Problems—Multimodal Single-Cell Integration” hosted by Kaggle last year [103]. More multi-omics integration tools with the ability to handle time/spatial dimensions will become available in the foreseeable future.

As more datasets are curated, the computational burden and storage requirement will continue to increase and become impractical for operating in an on-site environment. Fortunately, cloud-based services are becoming increasingly popular and have started alleviating these burdens. However, this approach may not be widely adopted by the research community due to the lack of funding support, training, and/or other logistic concerns. Scaling will also be challenging. The multi-omics datasets are intrinsically large, especially the single-cell omics data, which significantly increase model complexity and computing time. Application of dimensionality reduction and representation learning methods such as autoencoders can help mitigate the issue but at the risk of losing information during the process. Finally, findings from in silico analyses should be validated in biological settings, which may be overlooked when using a comprehensive and powerful framework.

## The Era of Big Data and Precision Medicine

As high-throughput multi-omics data and healthcare data become increasingly accessible and artificial intelligence constantly makes breakthroughs, a new era of more personalised and effective medicines is likely to emerge [104]. Machine learning has been used to develop diagnostic, prognostic, and predictive tools from healthcare data [105, 106], and linking in multi-omics data will further drive the discovery of novel biomarkers, new drugs, and personalised preventative measures and therapeutic regimens. Analysing multi-omics data using machine learning is still at an early stage even though some of the techniques have already been explored for single omics data analysis [107, 108]. Mamoshina and colleagues applied multiple machine learning methods including support vector machines, random forest, and neural networks on muscle transcriptomics data and trained age predictors to predict human skeletal muscle age. They identified key genes and pathways including PPAR signalling and neurotransmitter recycling to be associated with skeletal muscle ageing via feature importance ranking [107]. Yang et al. followed the BERT (bidirectional encoder representations from transformers) approach in the natural language processing field; developed a pretrained deep neural network–based model, single-cell BERT, extensively bench-marked; and demonstrated to be superior at cell-type annotation, novel cell type discovery, and overcome batch effects in single-cell transcriptomic data analysis [108]. The nature of multi-omics data, including high sparsity, more features than observations, and the need for integration, currently poses unique challenges to machine learning applications [109]. However, as machine learning algorithms keep evolving, combining multi-omics and clinical data to inform healthcare decisions will become “the new normal”.

## Conclusion

Single-cell profiling has revolutionised biomedical research and fuelled many new discoveries in the cardiovascular field. Multiple single-cell transcriptomic atlases have been established in different species, providing valuable high-quality references for future studies. Major cell subtypes in the healthy and diseased hearts have been identified and their interactions charted. Studies of injury models across species have provided insights into shared mechanisms of myocardial regeneration, including the cell populations involved and the relevant signalling pathways. Comparative investigations have identified regenerative programs unique to the developing hearts that are not detected in the adult heart. Therefore, to now assimilate these insights efficiently and effectively from individual studies to inform new therapeutic strategies, we propose a multi-species, multi-omics, meta-analysis framework to constantly generate, evaluate, and prioritise new targets for cardiovascular regeneration. With the growing application of machine learning and artificial intelligence, we envision that linking and analysing healthcare and multi-omics data using machine learning will lead to a new era for cardiovascular regeneration.

## Data Availability

Not applicable.
